# Reproduction in Risky Environments: The Role of Invasive Egg Predators in Ladybird Laying Strategies

**DOI:** 10.1371/journal.pone.0139404

**Published:** 2015-10-21

**Authors:** Sarah C. Paul, Judith K. Pell, Jonathan D. Blount

**Affiliations:** 1 Centre for Ecology & Conservation, College of Life & Environmental Sciences, University of Exeter, Penryn Campus, Cornwall, United Kingdom; 2 J. K. Pell Consulting, Luton, Bedfordshire, United Kingdom; Federal University of Viçosa, BRAZIL

## Abstract

Reproductive environments are variable and the resources available for reproduction are finite. If reliable cues about the environment exist, mothers can alter offspring phenotype in a way that increases both offspring and maternal fitness (‘anticipatory maternal effects’—AMEs). Strategic use of AMEs is likely to be important in chemically defended species, where the risk of offspring predation may be modulated by maternal investment in offspring toxin level, albeit at some cost to mothers. Whether mothers adjust offspring toxin levels in response to variation in predation risk is, however, unknown, but is likely to be important when assessing the response of chemically defended species to the recent and pervasive changes in the global predator landscape, driven by the spread of invasive species. Using the chemically defended two-spot ladybird, *Adalia bipunctata*, we investigated reproductive investment, including egg toxin level, under conditions that varied in the degree of simulated offspring predation risk from larval harlequin ladybirds, *Harmonia axyridis*. *H*. *axyridis* is a highly voracious alien invasive species in the UK and a significant intraguild predator of *A*. *bipunctata*. Females laid fewer, larger egg clusters, under conditions of simulated predation risk (P+) than when predator cues were absent (P-), but there was no difference in toxin level between the two treatments. Among P- females, when mean cluster size increased there were concomitant increases in both the mass and toxin concentration of eggs, however when P+ females increased cluster size there was no corresponding increase in egg toxin level. We conclude that, in the face of offspring predation risk, females either withheld toxins or were physiologically constrained, leading to a trade-off between cluster size and egg toxin level. Our results provide the first demonstration that the risk of offspring predation by a novel invasive predator can influence maternal investment in toxins within their offspring.

## Introduction

Maternal fitness is increased by maximising the number of offspring that survive to reproduce [1, 2]. As the resources available for reproduction are finite, there is a trade-off between fecundity and per-offspring maternal investment [3, 4]. Classically egg size has been used to identify this trade-off [5–7], however, while egg size may often be a good proxy for maternal investment, there are exceptions [8, 9]. In some cases measuring components of egg composition, e.g. hormones, carotenoids or other micronutrients that may influence offspring fitness [10], can be a more accurate representation of per-offspring maternal investment [11, 12]. Egg chemical defence is one such component that can influence offspring survival [13], and is particularly important in species with no or little parental care, such as many insect species [14]. However, it can be costly [15], both metabolically, with costs associated with toxin production and storage [16, 17], and if sequestered from the environment, where costs are associated with foraging for the toxins themselves [18]. Therefore, trade-offs may exist between egg toxin level and the size and number of offspring, but this remains to be tested.

Optimal per-offspring investment is also dependent on the reproductive environment; that is the quality of the environment into which the offspring will emerge [19]. To maximise offspring survival in poorer quality environments, the optimal investment will be larger than in environments with more favourable conditions [20, 21]. Where reliable cues about the nature of the offspring environment exist, mothers can adjust offspring phenotype in order to maximize offspring survival. Such ‘Anticipatory Maternal Effects’ (hereafter AMEs; [22]) involve an increase in maternal fitness through a concomitant increase in offspring fitness [23, 24] and examples of predator-driven AMEs have been identified across multiple taxa [25–29]. For selection to favour AMEs, the maternal environment at the time of reproduction must be a good predictor of the environment that her offspring will experience, and the cost of plasticity must be outweighed by the increase in maternal fitness accrued through the change in offspring phenotype [23].

Studies of AMEs, and of maternal effects in general, focus heavily on natural environmental variation, for instance fluctuations in food abundance and the aforementioned predation risk [23, 30]. This makes sense as it is adaptations to these natural environmental fluctuations and perturbations that will have been selected for over the course of a species evolutionary history [31]. However, modern day ecosystems are currently experiencing dramatic, anthropogenically driven change, for example from pollution, land use change, pesticide use, invasive species and climate change [32, 33]. Maternal effects are a powerful mechanism by which females can respond to this change and consequently should be considered when assessing the impact of any of these anthropogenically driven factors on species and populations. For instance the priming of offspring phenotype to increases in temperature, drought and heavy metal abundance, via maternal exposure to these factors has been demonstrated in plants and a species of butterfly [34–36]. Furthermore alterations in the maternal environment, induced by anthropogenic change, may also have indirect beneficial effects on offspring fitness, again mediated by maternal effects. One such case is found in *Daphnia magna* where offspring produced by mothers reared at higher temperatures had lower susceptibility to disease than offspring of control mothers [37]. Unlike pollutants and climate change there has been little focus on the way maternal effects may mediate the impact of invasive species on natives. This is surprising considering the increase in the number and global spread of invasive species in recent decades [38, 39], and their well-documented negative impact on the fitness of native species, e.g. via predation of offspring [40]. Consequently, determining how females modulate investment, via maternal effects such as AMEs, in the face of such novel offspring predators, is crucial in order to understand the complex effects of invasives on native species.

Conspicuous and chemically defended (aposematic) ladybirds are ideal species in which to investigate the reproductive strategies of females in environments with variable levels of offspring predation risk, by an invasive predator. Such ladybirds show no maternal care and lay clusters of brightly coloured eggs that are chemically defended by autogenously produced alkaloids [41]; known to be a costly form of defence in adults [42]. These alkaloids are present in the tissue and on the surface of ladybird eggs [43, 44], and (between- and within-maternal) variation in egg alkaloid levels affects egg predation rates [45]. The eggs have numerous predators [46, 47], including the larvae of invasive ladybird species [48–50]. The presence and abundance of such predators varies greatly in space and time [51, 52], meaning that optimal toxin investment can vary between reproductive environments. Females delay the onset of egg laying and lay fewer eggs in response to chemical cues that reliably indicate the presence and abundance of larval predators [53–56]. Furthermore, egg clustering deters predation by heterospecific larvae [57]. However, whether females modulate toxin investment in eggs, considering the high potential costs of toxin production, or cluster size in response to predation risk remains unknown.

We investigated the effects of simulated predation risk on the egg laying behaviour of ladybirds including their investment in egg toxins. Two-spot ladybirds, *Adalia bipunctata*, were allowed to lay eggs in environments that either contained larval tracks of harlequin ladybirds, *Harmonia axyridis*, (P+) or that contained no tracks as a control (P-). *H*. *axyridis* is an invasive species in the UK, and being highly polyphageous and competitive, it poses a serious risk to *A*. *bipunctata* populations in the wild [58,59]. Eggs of *A*. *bipunctata* contain the alkaloid adaline [60] and we predicted that females in P+ conditions would lay eggs that contained higher adaline concentrations compared to females in P-, control, conditions and that consequently there would be a trade-off between egg number and egg toxin level. As egg clustering deters predation by heterospecific ladybird larvae [57], we also predicted that larger individual clusters of eggs would be laid under P+ conditions than under P- conditions. Finally we predicted that P+ females would delay egg laying (increased latency) and produce fewer eggs overall than P- females, in agreement with previous studies [53, 61, 62].

## Materials and Methods

A stock culture of *A*. *bipunctata* (f. *typica*), obtained from Syngenta Bioline (Little Clacton, Essex, CO16 9QG), was maintained in a cage on an *ad lib*. diet of pea aphids, *Acyrthosiphon pisum*, at 20°C in a 16L:8D h photoperiod. The *A*. *pisum* prey were reared in cages on dwarf bean (*Vicia faba*) under the same abiotic conditions as the *A*. *bipunctata*. Experimental individuals of *A*. *bipunctata* were 1st generation virgin adults of known age (20–25 days post eclosion) reared from individuals obtained from the stock culture: 44 females and 44 males from five different adult pairs. Each female was mated with a non-sibling male and after 1h females were removed and placed individually into an experimental Petri dish that differed in simulated predation risk (see below) and provided with *A*. *pisum ab lib*. Females from different sibling clusters were distributed evenly between the two treatment levels, so that family ID and mate ID were represented equally in both P+ and P- treatments. Family ID refers to the adult pair from which the experimental females were reared i.e. the identity of their parents, and mate ID to the identity of the parents (i.e. adult pair) from which experimental males were reared. Experiments were carried out in an incubator (Percival^®^ model I-41LL, 505 Research Drive, Perry, IA 50220 USA) at 18°C and a 16L:8D h photoperiod.

To create an environment that conferred a simulated risk of predation (P+), 4th instar *H*. *axyridis* larvae were placed, without food, into individual sterile Petri dishes (9cm diam.), each containing a semicircle of corrugated filter paper (9cm diam.) and left for 24 h [53, 63], after which time they were removed. A control environment of no simulated predation risk (P-) consisted of a sterile Petri dish (9cm diam.) and a clean semicircle of corrugated filter paper that had not been in contact with *H*. *axyridis*. Mated *A*. *bipunctata* females were placed individually into a P+ or P- Petri dish, with adlib *A*. *pisum* and the number of eggs and individual clusters of eggs laid was recorded at 1, 3, 6, 9, 12 and 24 h intervals. Ad. lib *A*. *pisum* were provided to reduce the risk of filial cannibalism [64], additionally dishes were monitored for evidence of cannibalism, easily detected through the presence of egg remains, and females were excluded from the analysis if cannibalism had occurred. After 24 h females were removed and, along with all clusters of eggs laid, frozen at—80°C prior to chemical analysis. A cluster was classified as a group of two or more eggs, with each egg being in physical contact with at least one other egg in that cluster. Each cluster was frozen individually and, depending on cluster size, one to six eggs were randomly selected from each cluster laid by each of the females, with the exception of one female where only one of the two clusters of eggs laid was analysed. These eggs were weighed to the nearest 0.1μg, individual egg weight is referred to as egg mass from this point onwards, and alkaloid (adaline) levels analysed.

### Quantifying levels of adaline

Each egg was weighed to the nearest 0.1μg using an XP6U Ultra-microbalance (Mettler-Toledo) and homogenized using a hand held pestle (Fisherbrand™ Pellet Pestle™ Cordless Motor) for 30 s in 200μl chloroform with an internal standard of 1ng/μl E,Z-4,7 tridecadienyl acetate (Pherobank, 6700 AH Wageningen). Samples were then centrifuged at 17.7 x g for 3 min, and an aliquot (100μl) transferred into an autosampler vial. Similarly for adults, the elytra, which unlike the body tissue are purely structural (keratinous) and contain no, or undetectable levels of alkaloids [65,66], were removed and the body was weighed to the nearest 0.01mg using an analytical balance (GR-200 A&D^®^ Gemini™) before being homogenised for 60 seconds in 500μl chloroform with an internal standard of 1ng/ μl E,Z-4,7 tridecadienyl acetate. After homogenization a second 500μl of solvent solution was added. Each sample was then centrifuged at 17.7g and 13.3rpm for 3 minutes. 10μl of extract solution and 90μl of solvent solution was then transferred into an autosampler vial. Samples (2μl) were injected into an Agilent 7890A GC coupled with a 5975B MS fitted with an HP5-ms column (30mx0.25mmx0.25μm film thickness). The injection was in pulsed splitless mode, and the inlet temperature was 250°C. The carrier gas was helium with a flow rate of 1.3 mL/min. The GC temperature programme was 50°C at injection increasing to 140°C at 20°C/min, then from 140°C to 280°C at 5°C/min. Mass spectra operated in SIM mode, scanning for ions m/z (166.2 for Adaline) and (79. 1 for standard). Adaline (ng/mg body tissue) was quantified relative to the internal standard.

### Data analyses

All analyses were carried out using R version 3.0.2 (R Development core team, [67]). Data were examined for normality, homoscedasticity and outliers. The alpha level was set at 0.05 for all tests and stepwise backwards deletion was employed to reach the minimum adequate model [68]. A multinomial logistic regression model (package = mlogit) was fitted to ascertain whether there was a difference in the onset of laying between the two treatments, i.e. if the presence of *H*. *axyridis* tracks deterred laying.

A general linear model (package = MASS, function = glm) was fitted to the sqrt of total egg number with treatment, total cluster number and female mass (mg) as covariates. Generalized linear modelling (package = MASS, function = glm, family = quasipoisson) was used to identify differences in both the total cluster number and mean size of clusters per female between treatments, with total egg number and total cluster number as respective covariates and female mass (mg) as a covariate in both models.

There was statistically significant repeatability of egg mass, the weight (mg) of individual eggs, and egg adaline concentration within clusters (Egg adaline: R = 0.749, SE = 0.042, CI = 0.656, 0.816, P = 0.001; Egg mass: R = 0.599, SE = 0.055, CI = 0.472, 0.69, P = 0.001) and females (Egg adaline: R = 0.750, SE = 0.057, CI = 0.609, 0.832, P = 0.001; Egg mass: R = 0.528, SE = 0.068, CI = 0.379, 0.642, P = 0.01). Repeatability was calculated using a generalized linear mixed effects model with a log link for egg adaline and a linear mixed effects model for egg mass in the ‘rptR’ package following [69, 70]. These results supported the use of a subsample of eggs from each cluster as representative of the adaline and mass of eggs per female.

Variation in egg adaline concentration (ng/mg) with treatment, maternal adaline concentration, total egg number or mean cluster size, and a two way interaction between treatment and total egg number/mean cluster size was assessed using generalised mixed effects modelling (package = lme4 [71]), function = glmer, family = poisson) with female and cluster identity as nested random effects. Variation in egg mass (mg) with treatment, female mass (mg), total egg number or mean cluster size, and a two way interaction between treatment and total egg number/mean cluster size was assessed using linear mixed effects modelling (package = lme4 [71]), function = lmer) with female and cluster identity as nested random effects. Models were simplified using a backwards stepwise deletion approach [64] and results are reported for all main effects and significant interactions (P< 0.05).

There was no difference between the two treatments in whether or not a female cannibalised her eggs (Chi-Sq; X^2^_1_ = 2.530, P = 0.112). However, the specific number of eggs cannibalised could not be quantified, and therefore only females that did not cannibalise their eggs were included in the analyses (n(Fem) = 28 and n(Cluster) = 49).

## Results

**Fig 1:**
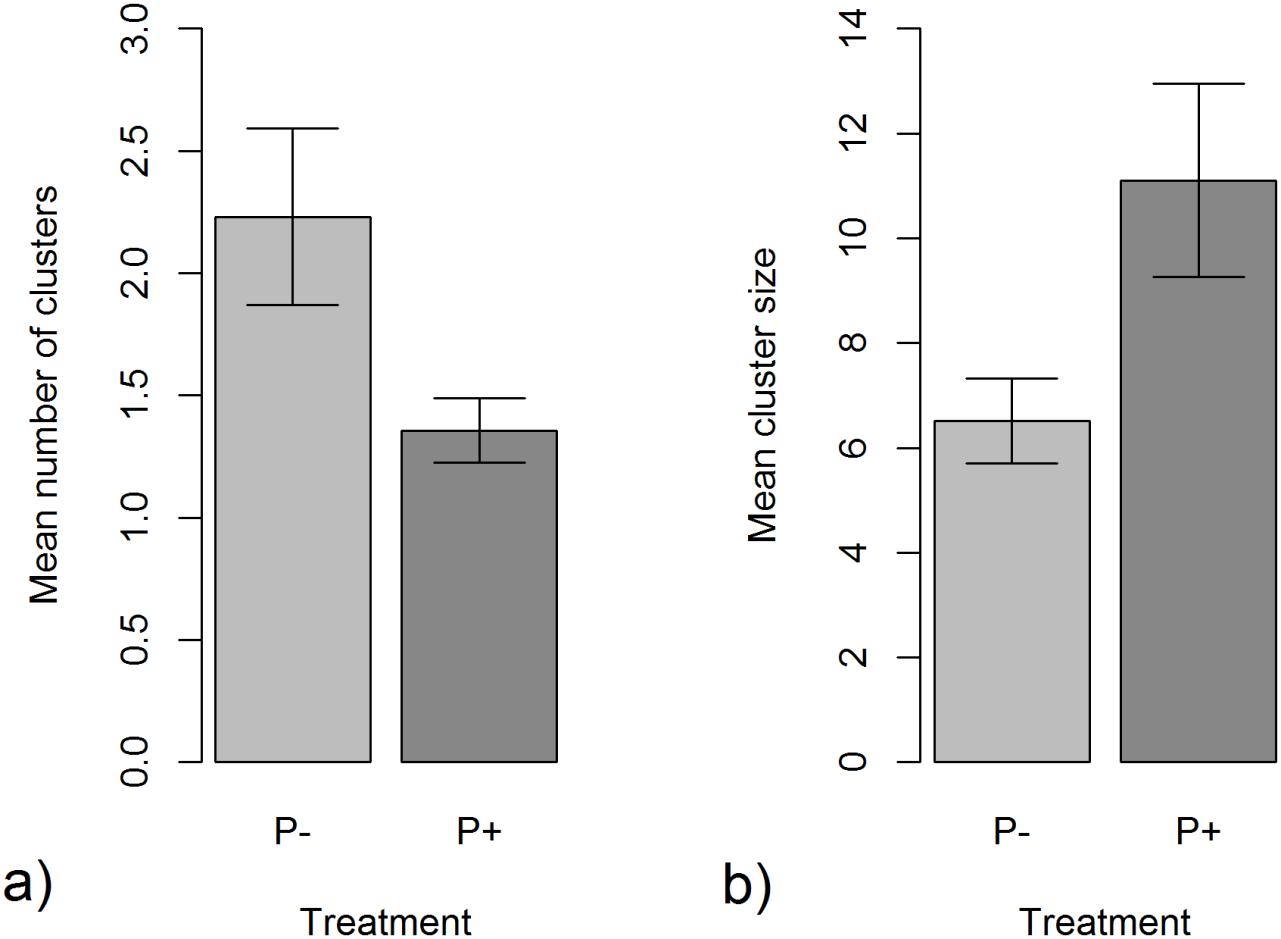
a) mean (± SE) number of clusters laid per female and b) mean size (± SE) of clusters laid per female under conditions of either no predation risk (P-) or simulated predation risk (P+).

The latency period before egg laying started did not differ significantly between P- and P+ groups (X^2^_1_ = 4.236, P = 0.30, R2 = 0.058; (P-): 17 ± 2 h, (P+):15 ± 2 h (mean time till first egg laid ± SE)). Similarly, the total number of eggs laid by females did not differ significantly between the P- and P+ groups (F_1,24_ = 0.6965, P = 0.413). However, the pattern of laying did differ; in the simulated presence of predators (P+) the total number of clusters laid was significantly smaller (Fig 1a; X^2^_1,24_ = 7.554, P<0.01), but the mean cluster size was greater (Fig 1b; X^2^_1,24_ = 4.826, P = 0.03) than when predator cues were absent (P-).

Though there was no treatment effect (see above) egg mass, the weight of individual eggs (mg), significantly increased with both mean cluster size (mean cluster size, X^2^_1_ = 4.363, P = 0.036; treatment*mean cluster size, NS) and total egg number (total egg number, X^2^_1_ = 3.950, P = 0.047; treatment* total egg number, NS).

**Fig 2:**
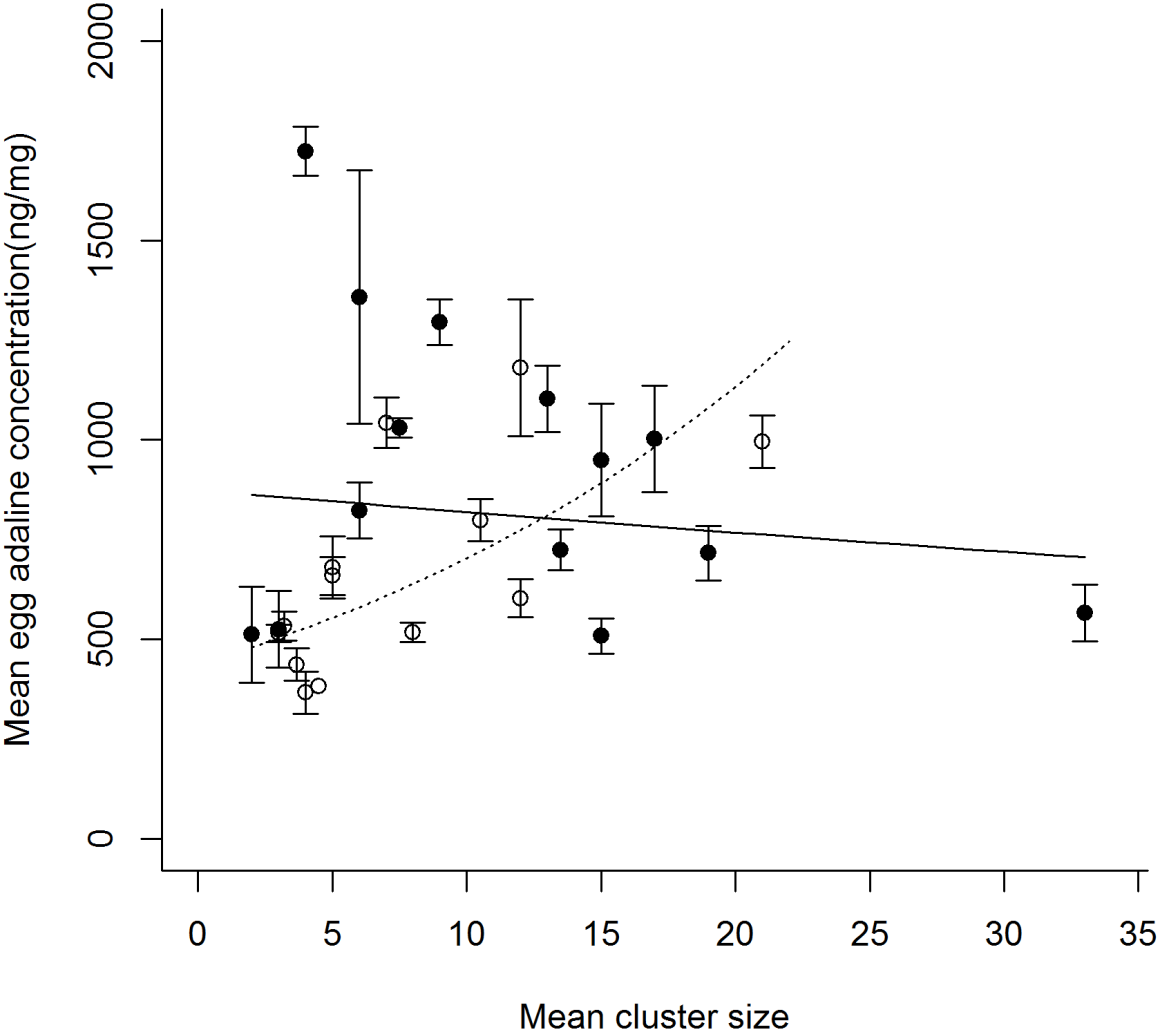
Mean egg adaline concentration (ng/mg, + SE) in relation to mean cluster size, per female under conditions of either no predation risk (◌, ---) or simulated predation risk (●,─). Trend lines are back transformed predictions from glmm controlling for effects of female and cluster ID.

The concentrations of adaline (mg/ng) in adult females and their eggs were not significantly correlated (X^2^_1_ = 1.044, P = 0.307). Adaline levels did not differ significantly between treatments (X^2^_1_ = 1.867, P = 0.172) and were not correlated with egg number (total egg number, X^2^_1_ = 0.225, P = 0.636; treatment*total egg number, NS). However, there was an interactive effect of treatment and mean cluster size on egg adaline levels (X^2^_1_ = 6.428, P = 0.012); there was a positive relationship between egg adaline concentration and mean cluster size for P- females, whereas the opposite pattern was found for P+ females (Fig 2).

## Discussion

Simulated predation risk did not affect either the number of eggs laid by females or the time at which they began to lay eggs. However, the way in which eggs were distributed amongst clusters did, with females laying fewer larger clusters under conditions of simulated predation risk than when predator cues were absent. The mean size of clusters laid by females was positively correlated with egg mass irrespective of treatment. Additionally, under conditions free from predation risk, there was a positive relationship between mean cluster size and egg toxin level whereas, in contrast, under conditions of simulated predation risk the slope of the relationship between mean cluster size and toxin level was negatively signed.

The positive relationship between mean cluster size and egg adaline levels under P- conditions indicates that, in a risk-free environment, cluster size could be a ‘quantitatively honest’ signal of egg toxin level, where, in relation to defence against predators, stronger or more conspicuous signals are associated with better defended individuals [72]. Such signalling honesty is thought to be maintained by the differential costs and benefits of signalling (handicap principal; [72, 73]) where either: stronger signallers suffer more attacks but lower mortality than weaker signallers due to predator rejection after handling prey (‘go slow’ mechanism; [74]); or physiological coupling between the signal and the defence selects for stronger signallers to suffer fewer attacks and lower mortality than weaker signallers (resource allocation model; [17]). In the case of cluster size either mechanism could be involved. The size of the cluster itself may send a stronger or more ‘efficient’ deterrent signal to predators, either chemically or visually, as demonstrated by the aggregation of aposematic individuals [75]. This in turn may cause predators to be cautious and ‘go slow’ when attacking larger clusters, the eggs of which they are more likely to reject, thus increasing the survival of eggs in larger clusters. Alternatively, eggs are expensive to produce [76, 77] as are toxins [42], and so increasing cluster size would be likely to involve a concomitant decrease in egg toxin level due to the finite resources available [17]. Models have demonstrated that such resource allocation trade-offs between signal and defence can lead to an evolutionary stable strategy where individuals allocate resources optimally between defence and signalling, resulting in a positive correlation between the two [17, 72]

In contrast, the negatively signed slope under P+ conditions suggests that, in the presence of predator cues, signal honesty broke down and cluster size was no longer a reliable signal of egg toxin levels. We suggest three possible explanations for the negative relationship between mean cluster size and egg toxin level under conditions of simulated predation risk (P+). Firstly, it is possible that P+ females withheld investment in costly toxins [42] as an example of ‘selfish maternal effects’ (from now on SMEs) [23]. Though increased *A*. *bipunctata* egg toxin levels have been linked to reduced consumption by predators [78], *H*. *axyridis* larvae are voracious, have high tolerance of novel alkaloids [79, 80] and show limited discrimination between eggs of varying toxicity [81]. Consequently, modulation of toxin investment in eggs may not alter egg survival prospects in the face of this particular predator. It may therefore be more beneficial to withhold investment, in order to conserve resources for future reproductive events in a potentially less risky environment [23], a strategy also shown by females in other taxa after mating with poor quality males [82, 83]. If P+ females were withholding investment, however, a reduction in the mass and total number of eggs laid may also be expected, compared with P- females, but this was not found.

Secondly, as cluster size ‘honestly’ signalled egg defence under P- conditions it is possible that under P+ females laid larger clusters to increase perceived levels of egg defence and therefore reduce predation risk, in an act of intraspecific Batesian mimicry or automimicry [84]. Theoretical and empirical work has demonstrated that low levels of such automimicry can persist in populations [85–89]. ‘Cheats’ (aka automimics) benefit from assuming the signal of better defended conspecifics and, though they degrade the ‘common good’, non-cheating conspecifics are still more likely to survive predation attempts due to their higher levels of defence and therefore unpalatability [90]. Speed and Franks [91] have also recently argued that automimicry rather than reaching a stable equilibrium between cheats and non-cheats persists in populations as a result of antagonistic co-evolution, which leads to an evolutionary chase between individuals with poor levels of chemical defence and individuals with high levels of chemical defence. The result is a mixture of ‘honest’ and ‘dishonest’ signallers within the population, depending on the co-evolutionary cycle’s progress.

Though frequently used to explain the diversity of defence and associated warning colouration seen in aposematic populations [92], automimicry may also apply to other visual signals, such as cluster size. It is however, worth noting that ladybird eggs are aposematic [93] and a component of any deterrent signal given by larger cluster size may not merely be a property of the size of the cluster itself but also of its conspicuousness. Aggregation of aposematic individuals improves predator deterrence by increasing the efficiency of the aposematic signal [75, 94]. Conspicuousness as well as cluster size may, therefore, be an important component of signalling the toxin level of eggs in a cluster, an important consideration for future work.

What is not immediately obvious, is why laying larger clusters, as seen under P+ conditions, would be associated with a decrease in toxin level, i.e. why did females under P+ conditions cheat? One explanation is that a physiological trade-off between cluster size and egg toxin level became manifest in P+ females and not P- females, as the former laid significantly larger clusters than the latter. Alkaloid toxins are energetically expensive to produce [14, 42], so there may have been a limit to the quantity of toxins females could produce per reproductive event. Therefore any increase in the number of eggs laid per discrete laying event, i.e. an increase in cluster size, may have reduced per egg toxin allocation. Examples of such physiological restriction in egg investment have been recorded previously, for example in lesser black-backed gull (*Larus fuscus*) eggs, where egg lipid content increased and yolk-to-albumen ratio decreased with increasing egg number [11, 95].

Thirdly, P+ females may have laid larger clusters for reasons other than automimicry of larger and more toxic clusters. Egg clustering by insects can decrease predation [96, 97] including predation of ladybird eggs by heterospecific larvae [57] and, in addition to stronger aposematic signals, the so called ‘avoidance’ and ‘dilution’ effects are thought to be key to this reduction in predation [98]. The avoidance effect is a reduction in the likelihood of a predator encountering a group or cluster of prey than an equal number of individual solitary prey [99]. Even if a predator then detects a prey aggregation it is also unlikely to be able to consume all of the prey, increasing the proportion of prey individuals that survive compared to an attack on fewer or lone prey, a.k.a. the ‘dilution’ effect [98, 100, 101]. Both effects can also counterbalance the effect of higher detection rates resulting from the aforementioned stronger deterrent signals produced by clusters [102]. Increasing egg cluster size under P+ conditions could therefore, have been an effective anti-predator strategy irrespective of changes in aposematic signal strength. Again the possible physiological cost of producing large clusters can be invoked here to help explain the concomitant reduction in egg toxin level with increasing cluster size under P+ conditions.

It is also worth noting that ladybirds can lay infertile eggs. This infertility can be caused by STIs, such as Wollbachia sp. [103, 104], or be the result of trophic egg laying on the part of the female. Trophic eggs are infertile eggs laid by mothers in order to provide extra resources for their offspring [105]. The production of these eggs is an adaptation to poor resource conditions, and accordingly female ladybirds increase the number produced when laying in areas with low food availability [106]. As trophic egg production is strongly associated with low aphid numbers, variation in the number of infertile eggs between the two treatment levels would not be expected *a priori* as this experiment did not manipulate resource availability, providing adlib aphids prior to and during the experiment. However, it is interesting that intraguild predators such as *H*.*axyridis*, are not only a source of predation risk for offspring but also of competition for resources. The aphid colony will be being exploited, possibly heavily, by those ladybird larvae already present, and the more immediate risk of predation for offspring, will be superseded by low resource availability when offspring hatch. Females may therefore respond to intraguild predator presence by increasing the number of trophic eggs laid, another possible explanation for the larger clusters laid in the P+ treatment. The adaptive nature of such a strategy is however questionable as predatory larvae may consume the extra eggs. Additionally though previous trophic manipulation studies recorded changes in the proportion of trophic eggs per cluster there was no change in cluster size itself [106]. Therefore the evidence to support the occurrence of trophic egg laying in this experiment is weak, but cannot be ruled out as egg toxin analysis is destructive. Additional work could therefore be carried out to ascertain whether predation risk does affect trophic egg laying.

The lack of difference between the two treatment levels in both total egg number and latency to lay, contrasts with previous studies using *A*. *bipunctata*, where the presence of heterospecific predators or their tracks delayed the onset of laying [63] and egg number was reduced as a consequence [107]. This discrepancy may be because our experimental females were slightly older than in the previous studies [56, 108], though still at the age of peak fecundity [109], and only mated 24 hours prior to the experimental start point. As a result they were likely to have been time-limited rather than egg-limited [110] and therefore may have been less discriminatory than younger individuals about the environments in which they laid [111, 112].

## Conclusions

In conclusion our results are the first demonstration that maternal exposure to heterospecific predation risk can influence toxin investment into eggs. Females increased cluster size but not toxin investment in eggs in the face of offspring predation risk, and the concomitant decrease in egg toxin level can be explained either via: 1) a reduction in investment due to SMEs, or 2) physiological constraint, where increases in cluster size, due to either the benefits of a) ‘cheating’ or b) the avoidance and dilution effects, caused a decrease in toxin level. Further work should focus on disentangling these possible explanations via: maternal resource manipulation, to assess whether constraint or SMEs were responsible for the reduction in toxicity associated with increased cluster size under P+ conditions; by assessment of whether egg, and therefore cluster signal strength (either visual or chemical), are honest signals of toxicity and how this varies under different predation conditions; and by manipulation of the strength of the cluster signal (again either visual or chemical) in predation experiments using *H*. *axyridis*, to establish whether cluster size influences survival by increasing signal strength or by avoidance and dilution effects. Finally this is the first demonstration that maternal effects are involved in the reproductive response of a native species exposed to an invasive predator of their offspring and future work is required in order to explicitly test whether this response increases or decreases maternal fitness i.e. whether it is adaptive or a form of evolutionary trap [113].
